# Precise right hemihepatectomy for the treatment of hepatocellular carcinoma guided by fusion ICG fluorescence imaging

**DOI:** 10.7150/jca.41039

**Published:** 2020-02-10

**Authors:** Shunyu Yao, Luyuan Zhang, Jinliang Ma, Weidong Jia, Hao Chen

**Affiliations:** 1 Department of Hepatic Surgery, The First Affiliated Hospital of USTC, Division of Life Sciences and Medicine, University of Science and Technology of China, HeFei, 230001, China; 2 Anhui Province Key Laboratory of Hepatopancreatobiliary Surgery, HeFei, 230001, China; 3 Xiangya School of Medicine, Central South University, ChangSha, 410008, China

**Keywords:** Indocyanine, Green fluorescence imaging, Hepatocellular carcinoma, Precise, Hemihepatectomy

## Abstract

To evaluate the clinical significance of fusion indocyanine green (ICG) fluorescence imaging in precise right hemihepatectomy for the treatment of hepatocellular carcinoma (HCC). 47 patients with HCC who underwent right hemihepatectomy were retrospectively analyzed. 18 of them guided by fusion ICG fluorescence imaging (FIGFI) while 29 patients underwent conventional right hepatectomy without guidance. Compared to the patients with conventional treatment, the intraoperative blood loss of the patients with guided surgery was significantly less, and no transfusion and hepatic occlusion were performed during the operation. Liver function recovery faster in guided group. The incidence of postoperative complications is also lower, and the recurrence rate in one year is significantly reduced. ICG fluorescence range of 18 patients in liver surface was consistent with the ischemic line, and their postoperative liver cross-sections were clearly demarcation. There were no significant differences in the mean operation time, blood loss, postoperative hospital stays, cases of blood transfusion, complication rate, or postoperative peak volume of ALT and TB between positive or negative staining groups. Pathology results of all patients demonstrated HCC and negative margins, and microvascular invasion occurred in 8 cases. The average follow-up time of 18 patients was 16.7 months, and recurrence was found in 5 cases after surgery. FIGFI could guide the anatomical right hepatectomy with real -time increased radical rate, accuracy and safety for the treatment of HCC, and therefore showed a promising prospect.

## 1. Introduction

The core of anatomical liver resection is precisely defining the range of liver sections 1, 2. It is not easy to distinguish the color difference between the left and right hemipatic by just blocking the flow of blood into the right hemipatial blood on the hepatic surface marker tangent line, even inject methylene blue into the right portal vein to stain the right hemihepatic, therefore it is difficult to improve the precision of hepatectomy. In recent years, with the rapid development of molecular imaging technology, indocyanine green (ICG), as a near-infrared fluorescent dye, has been intensively applied in the field of liver surgery 3-6. By injecting ICG into the corresponding liver segment or the liver lobe, we are able to develop an ICG fluorescence imaging system (photodynamic eye) to guide precise hepatectomy. Moreover, intraoperative ICG fluorescence can achieve three-dimensional staining of the liver for more than 8 hours. The cross-section of the hepatectomy can be determined to guide the hepatic parenchyma separation in real time to improve the accuracy of the right hemihepatectomy 7-10. In this study, a retrospective observational approach was utilized to evaluate the clinical significance of ICG fluorescence fusion imaging (FIGFI) in guiding accurate right hemihepatectomy for HCC.

## 2. Materials and Methods

### 2.1 Clinical Data

#### 2.1.1 General Information

A total of 47 HCC patients undergoing elective right hemihepatectomy at the Department of Hepatic Surgery, The First Affiliated Hospital of USTC from July 2017 to December 2017 were studied. Among them, there are 18 patients with the median age of 52.8 (31-79), including 15 males and 3 females being treated with FIGFI. All patients had Child A liver function, and general clinical characteristics is shown in Table 1. The institutional Ethics Committee approval for the project was achieved before the study was started and was in compliance with the Helsinki Declaration. Written informed consent was obtained from all patients. The study was approved by the Hospital Ethics Committee (Ethics approval No. 2017 Lunxun No. 114).

Inclusion criteria: (1) Patients with HCC preoperatively combined with imaging examination and laboratory examination were confirmed with pathological examination. (2) Accord with right hemihepatectomy indications. (3) There was no restriction for patient's gender and age. (4) General condition is good, ASA grade I, liver function Child Grade A, adequate liver reserve function and residual liver volume. (5) Complete clinical data.

Exclusion criteria: (1) Patients with abnormal bile metabolism or excretion. (2) Patients that are allergic to phthalocyanine green or have iodine allergies. (3) Severe heart, brain, lung or kidney dysfunction.

#### 2.1.2 Surgical Operation

Take the right upper abdominal inferior costal incision, layer by layer into the abdomen, after gallbladder resection, dissecting the first porta hepatis, separation, ligation and separation of the right hepatic artery, portal vein right branch. The hepatic ligament was dissected, the third liver portal was dissected, and the short hepatic vein was ligated one by one. After second porta hepatis was dissected, the ligament of the inferior vena cava was dissected, the right hepatic vein was isolated extrahepatically, and the right hepatic vein was cut off by cutting the closure device.

**Figure 1:**
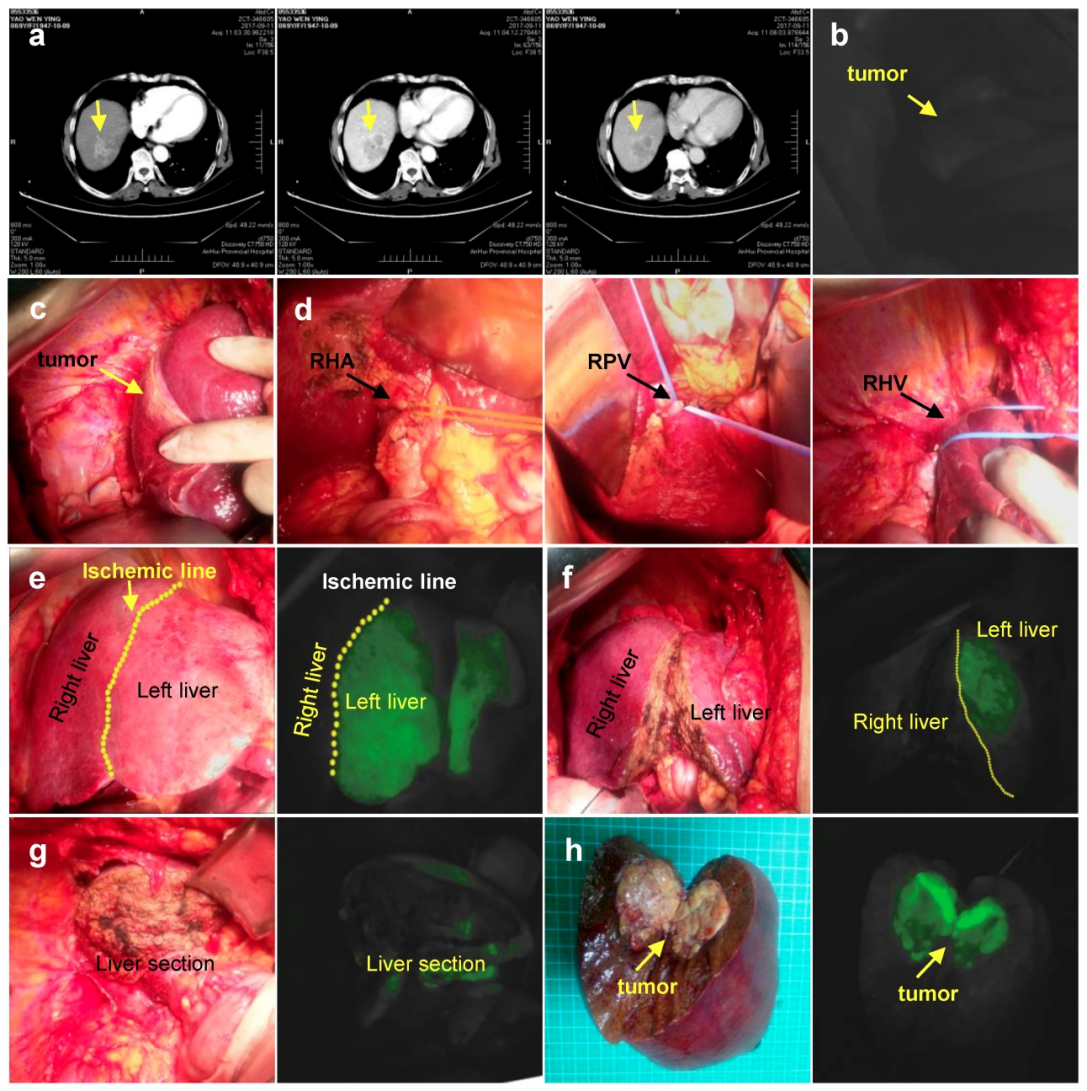
Negative staining.** a.** CT imaging data of tumor (Arrow). **b.** ICG can't detect tumor. **c.** Intraoperative exploration of tumor location. **d.** Ligation of the right hepatic artery, right portal vein, right hepatic vein in sequence. **e.** Left hepatic liver was gradually developed after 1 ml (2.5 mg/ml) ICG injected via peripheral vein.** f.** Liver resection under ICG navigation step by step. **g.** Complete resection of the right liver. **h.** Postoperative gross specimen and fluorescent display. RHA: Right hepatic artery; RPV: Right portal vein; RHV: Right hepatic vein

**Figure 2:**
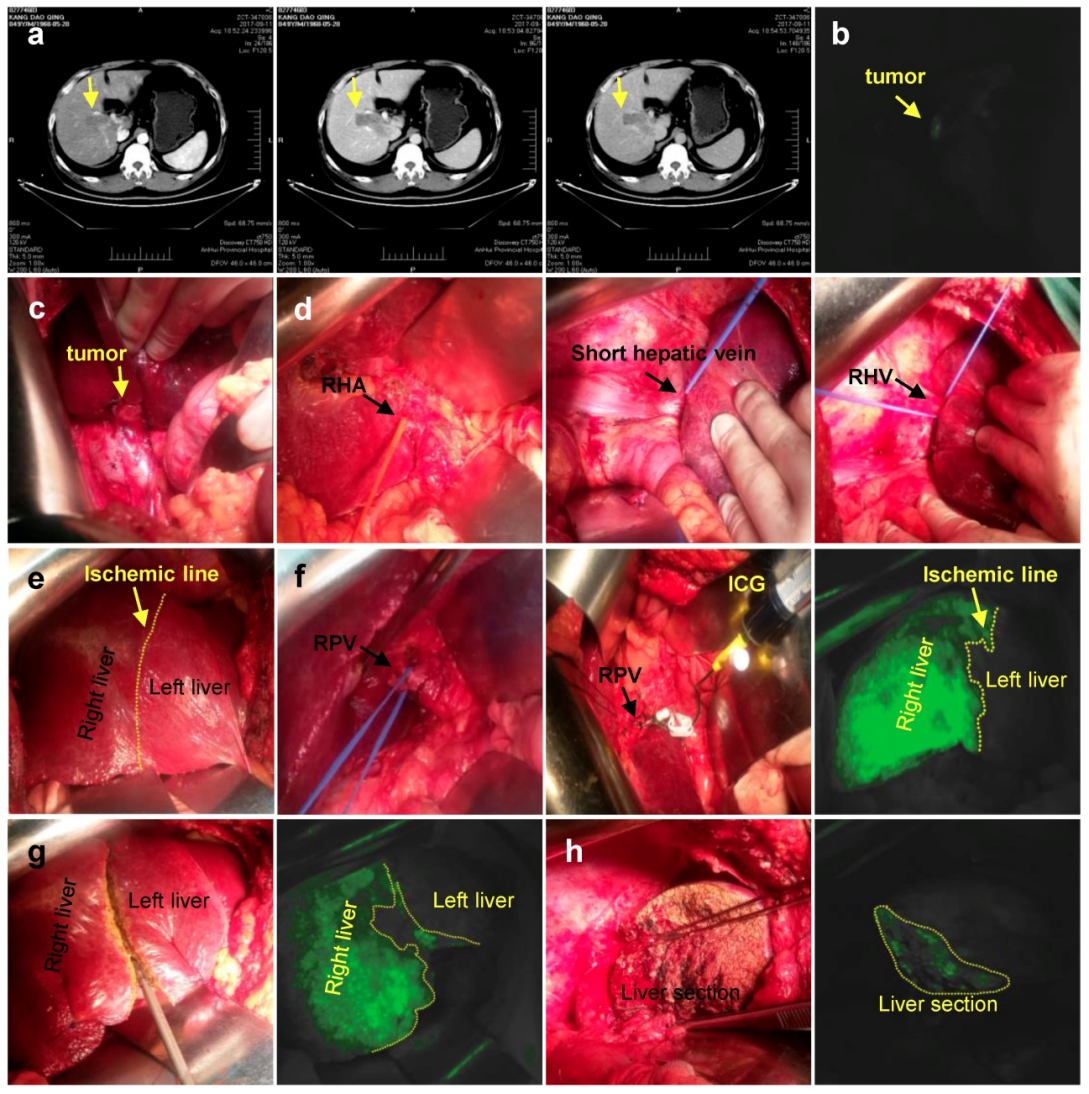
Positive staining. **a.** CT imaging data of tumor (Arrow). **b.** Intraoperative fluorescence display. **c.** Intraoperative exploration of tumor location. **d.** Ligation of the right hepatic artery, Short hepatic vein, right hepatic vein in sequence. **e.** Ischemic line after ligation of right hepatic vein. **f.** 1 ml (2.5 mg/ml) of ICG injected into the right branch of portal vein. **g.** Liver resection under ICG navigation step by step. **h.** Complete resection of the right liver. RHA: Right hepatic artery; RPV: Right portal vein; RHV: Right hepatic vein

Half-hepatic was developed using either negative or positive staining. (1) Negative staining: 1 ml (2.5 mg/ml) of ICG injected via peripheral vein (Figure 1); (2) Positive staining: 1 ml (2.5 mg/ml) of ICG injected into the right branch of portal vein (Figure 2). Then turn off the surgical lamp and place the probe perpendicular to the surface of the liver at a distance of 30-50 cm. See the left liver (20-30 s after ICG injection by the anti-display method) or the right liver (immediately after the ICG injection). The screen is gradually dark green, along the limits of the fluorescence can be marked on the liver surface resection line, ultrasonic knife was used of separate the liver parenchyma from shallow to deep following ICG fluorescence, which guided the clear display of the irregular middle fissure. Through the continuous opening and closing of surgical shadowless lights to judge and guide the direction of liver parenchyma off.

#### 2.1.3 Observation Indexes and Follow-up

(1) Intraoperative conditions: Results of right hemihepatectomy guided by FIGFI, whether the ICG fluorescence range is consistent with the ischemic line, operation time, intraoperative blood loss, and blood transfusions. (2) Postoperative recovery: postoperative hospital stays, peak postoperative ALT and TB, postoperative complications. (3) Postoperative pathological examination and follow-up: follow-up visits were conducted by outpatient and telephone methods. The follow-up contents generally included liver function, tumor index, and imaging examination (chest X-ray, abdominal ultrasound, CT, and magnetic resonance) to understand the patient. Postoperative recurrence and survival were followed up until December 2018.

### 2.2 Meta-analysis

#### 2.2.1 Literature Search Strategy

Computer search PubMed, EMbase, The Cochrane Library and CNKI database, and collect a cohort study on the application of ICG molecular fluorescence imaging technology in surgical treatment of liver tumors. The search time limit is from established to Aug 2019. The search terms are: indocyanine green fluorescence, ICG fluorescence, hepatectomy, liver cancer, liver tumor, liver neoplasm, hepatocellular carcinoma, hepatocellular cancer and hepatic neoplasm. All searches were conducted in a combination of subject terms and free words, and a second expanded search was conducted based on the references included in the study to improve the documented detection rate.

#### 2.2.2 Inclusion and Exclusion Criteria

The randomized controlled trial (RCT) published is preferred, and if there is no RCT, it is included in the cohort study. Participants: Patients with liver tumors undergoing elective hepatectomy were not limited in gender, age, race, and nationality. Interventions: The experimental group used ICG molecular fluorescence imaging technology to assist liver tumor resection, while the control group did not use. Outcome indicators: perioperative bleeding volume, operative time, blood transfusion rate, negative margin of cutting margin, postoperative complication rate, hospital stay. Exclusion criteria: (1) Non-Chinese and English literature. (2) Documents published repeatedly by the same research center or the same author. (3) The original research data could not be extracted and could not be obtained after contacting the author. (4) The control intervention group was not designed.

#### 2.2.3 Literature Screening and Data Extraction

Two researchers independently screened the literature, extracted the data and cross-checked. In case of disagreement, it is resolved through discussion or negotiation with a third party. The data extraction content mainly includes: (1) Basic information included in the research, including research topics, first author, region, published journal and time. (2) Study the key elements of design type and quality evaluation. (3) The basic characteristics of the subjects, including sample size, age, gender and tumor maximum diameter, Child-Pugh classification. (4) Specific details of the intervention. (5) Key elements of risk assessment for bias. (6) Outcome indicators and data of interest in each study.

### 2.3 Statistics Method

Clinical data were analysis by statistical package SPSS 13.0 (SPSS Inc., Chicago, IL). Continuous variables were expressed as the mean ± SE and were compared using Student's* t*-test. Categorical variables were compared using either the χ^2^ test or Fisher's exact test, as appropriate. A *P* value less than 0.05 was considered statistically significant.

Meta-analysis was performed using RevMan 5.3 software. The second categorical variable uses the odds ratio (OR) as the effect analysis statistic; the measurement data uses the mean difference (MD) as the effect analysis statistic, and each statistic calculates the 95% confidence interval (CI). The heterogeneity between the included studies was analyzed by *χ^2^* test, and the heterogeneity was determined by* I^2^* quantitative analysis: *P* >0.05, *I^2^* <50% was not statistically significant among the studies. *P* < 0.05 was considered statistically significant.

## 3. Results

### 3.1 Difference between patients with FIGFI guided and with conventional right hepatectomy

#### 3.1.1 Intraoperative conditions

There was no significant difference in the clinical parameters of the patients before surgery. And all of the patients were able to tolerate the surgery. The blood loss of guided group was significantly less than conventional group (261±144 ml *vs.* 438±384 ml, *P*=0.031), and no transfusion and hepatic occlusion were performed during the operation. The range of liver surface ICG fluorescence among 18 patients was consistent with that of the ischemic line. The postoperative cross-section was clearly demarcated. The operative time of 18 patients was 246 (150-345) min (Table 2).

#### 3.1.2 Postoperative recovery

**Figure 3:**
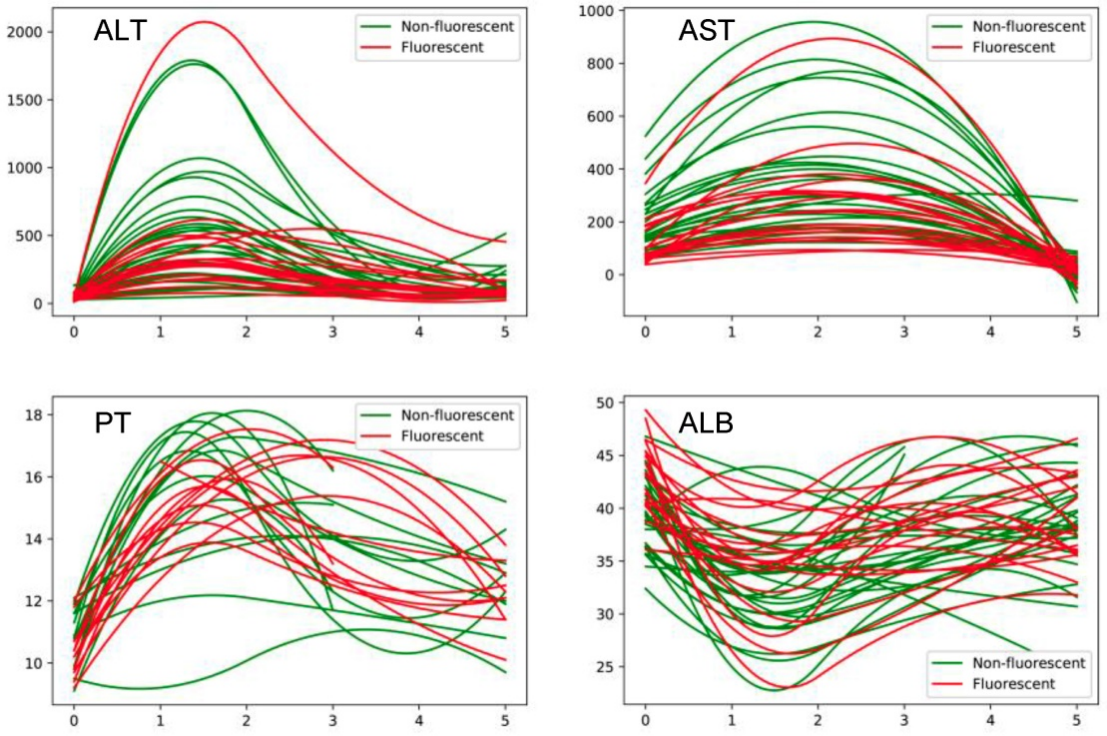
Perioperative changes in liver function between with and without ICG fluorescence navigation group.

The conditions of intraoperative and postoperative recovery of all patients are shown in Table 3. ALT maximum level was also lower in guided group, and we also detect the dynamic level of liver function (Figure 3). We found that the level of ALT, ALT and PT in guided group was significantly lower than conventional group by fitting the index of 1, 3, and 5 days after surgery. Meanwhile, ALB was lower in guided group. The mean postoperative hospital stay of guided group was (11.1±7.8) days, which is less than that of conventional group (13.4±7.1). The postoperative complication rate was 5/18 in guided group, comparing to 18/29 in conventional group (Table 3).

#### 3.1.3 Postoperative pathological examination and follow-up results

Navigation surgery did not result in an increase in costs. Postoperative pathological examination showed that the 18 patients in guided group were hepatocellular carcinoma with negative margins, including microvascular invasion in 8 cases. But there are 4 patients with positive margins in conventional group, which is a possible reason that the conventional group has the higher recurrence level and the worse outcomes (Table 4).

### 3.2 Difference between positive and negative staining of FIGFI guided right hepatectomy group

#### 3.2.1 Intraoperative conditions

Accurate right hemihepatectomy was performed under guidance of FIGFI in the 18 patients, of whom11 patients underwent reverse display while 7 patients underwent positive display as previously described (Table 5, 6). The range of the liver surface ICG fluorescence of total patients was consistent with that of the ischemic line. The postoperative cross-section was clearly demarcated. The operative time and blood loss of two groups did not have difference (Table 7). All patients did not receive blood transfusions.

#### 3.2.2 Postoperative recovery

There was no significant difference in the average time of hospital stay and liver function recovery after surgery (Table 8). In terms of complication rate, there was no significant difference between the anti-staining group 2/7 and the positive dyeing group 3/11. Because of the precise hepatectomy, the recurrence rate of guided group was significantly lower than that of the conventional operation group. There were 5 cases of recurrence after operation, which was related to preoperative tumor size and TNM classification.

### 3.3 Meta-analysis

#### 3.3.1 Document search process and results

**Figure 4:**
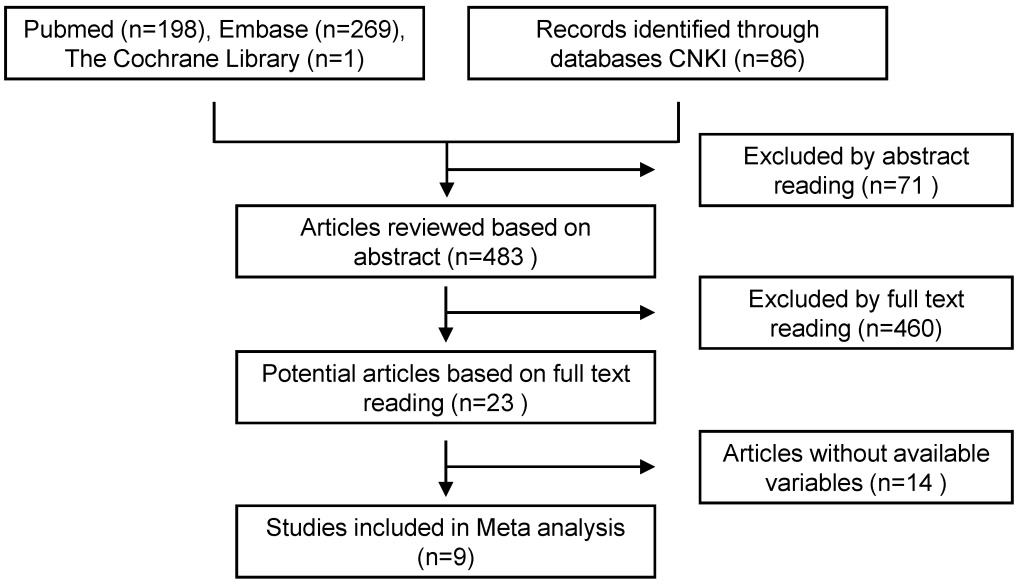
Literature screening flow chart

A total of 554 articles were included in the preliminary search, including 468 in English and 86 in Chinese. By carefully reading the topic and abstract, excluding duplicate and unrelated literature, 23 articles were included, and the full text was carefully read. According to the inclusion and exclusion criteria, 9 cohort studies were finally included (Figure 4, Table 9).

#### 3.3.2 Intraoperative indicators

##### 3.3.2.1 Perioperative bleeding volume

**Figure 5:**
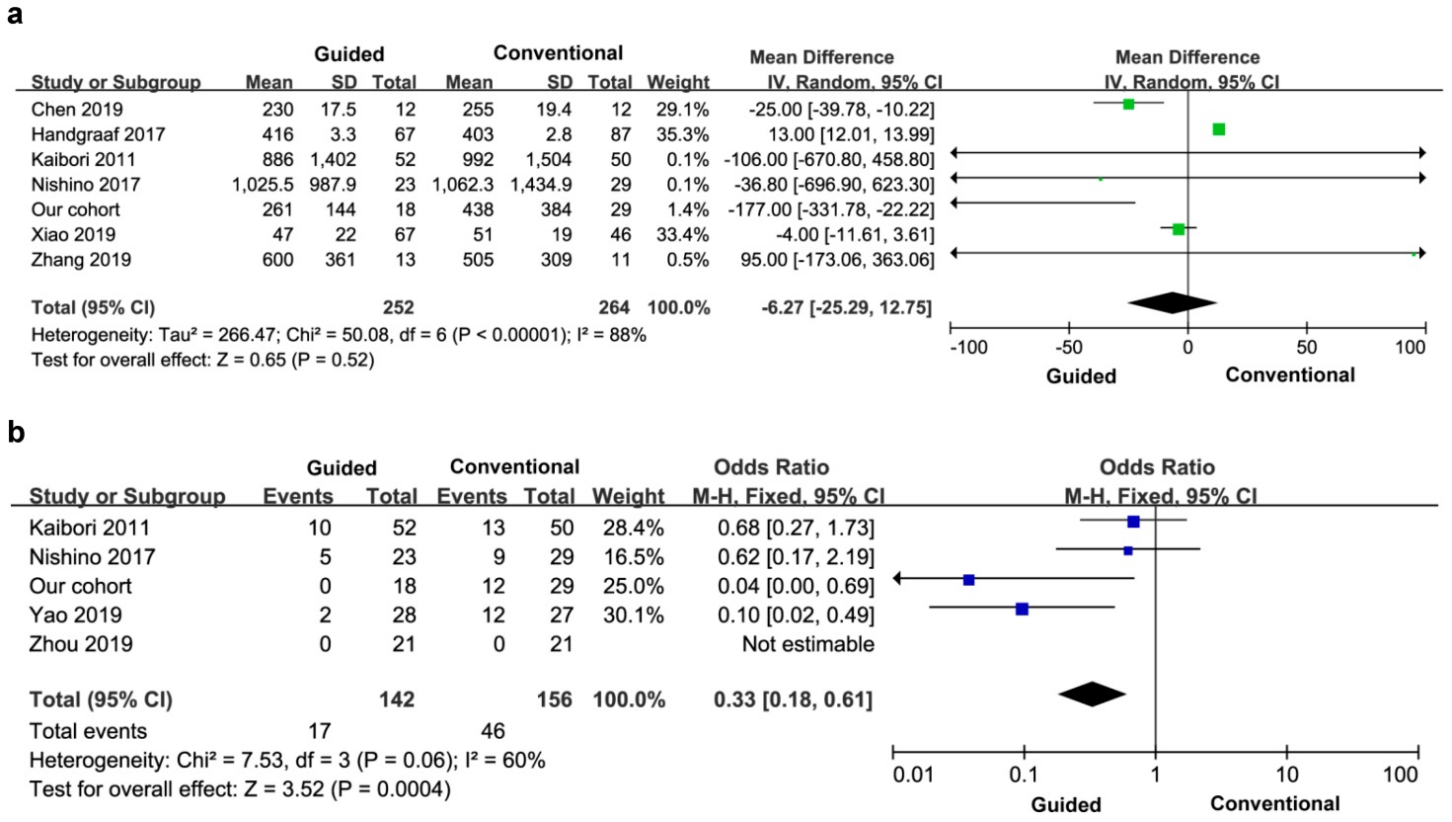
Perioperative bleeding volume **(a)** and blood transfusion rate **(b).**

Six studies reported perioperative bleeding, and three of the studies did not provide specific data, so the final study included seven studies and 516 patients. There was significant heterogeneity between the groups (I^2^=88%), so a random effects model was used. There was no significant difference in perioperative bleeding between the two groups (MD=-6.27, 95% CI=-25.29-12.75, *P*=0.52) (Figure 5a).

##### 3.3.2.2 Blood transfusion rate

Five articles reported the transfusion rate, a total of 298 patients. There was significant heterogeneity between the groups (I^2^=60%), and a fixed effect model was used. The blood transfusion rate of the experimental group was lower than that of the control group, and the difference between the two groups was statistically significant (OR=0.33, 95% CI=0.18-0.61, *P*=0.0004) (Figure 5b).

##### 3.3.2.3 Postoperative complication rate

**Figure 6:**
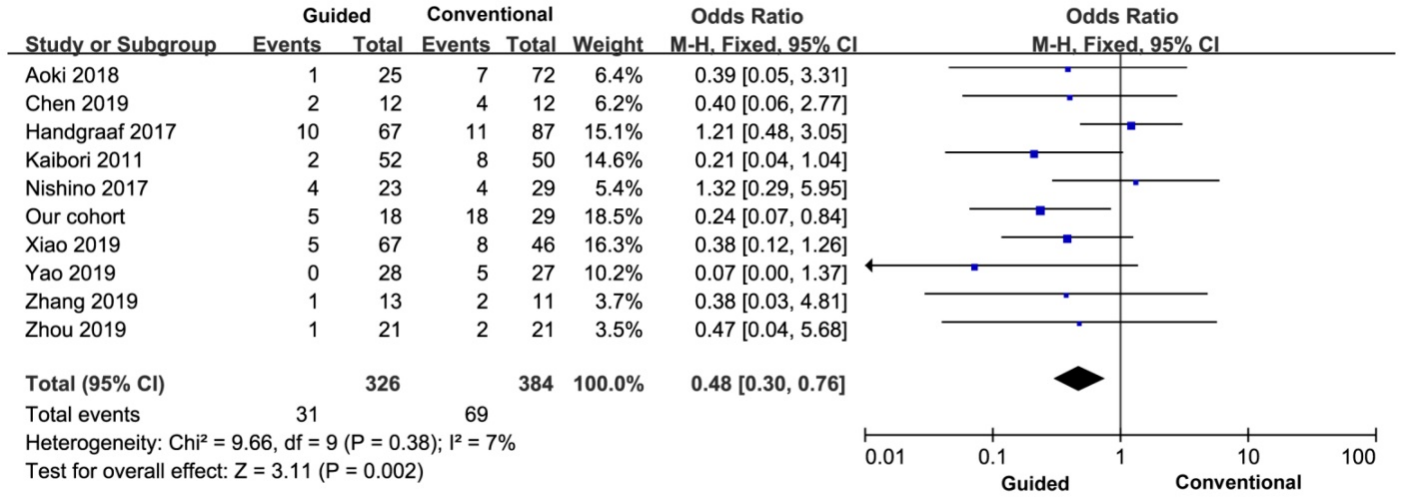
Comparison of postoperative complications

All the nine studies reported the incidence of postoperative complications, so the final study included ten studies and 710 patients. There was no heterogeneity between the groups (I^2^=7%), and a fixed effect model was used. The incidence of postoperative complications in the experimental group was lower than that in the control group, and the difference was statistically significant (OR=0.48, 95% CI=0.30-0.76, *P*=0.002) (Figure 6).

##### 3.3.2.4 Negative rate of margin

**Figure 7:**
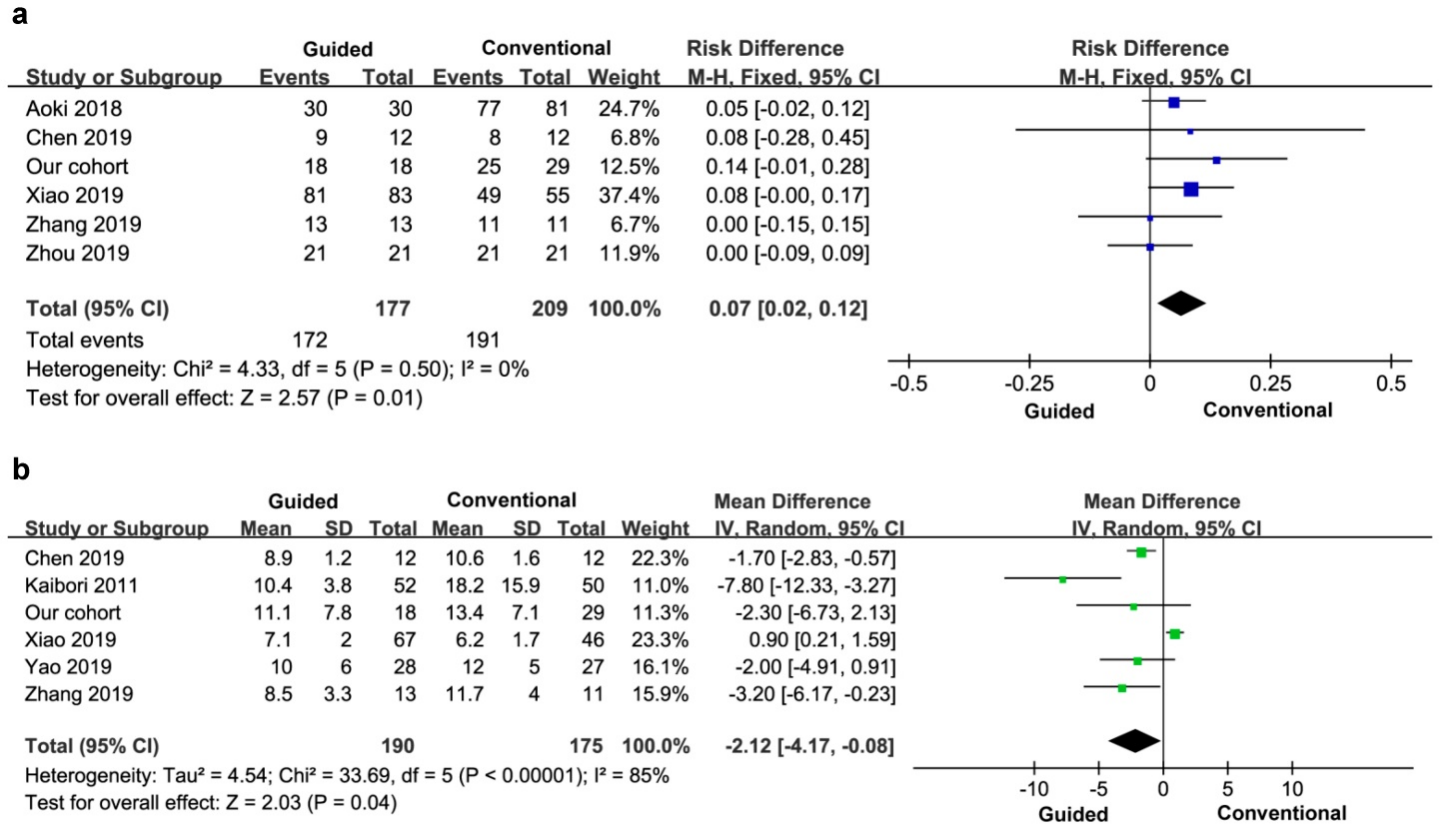
Postoperative negative margin **(a)** and hospital stay **(b)**.

Five cases reported a negative rate of margin, a total of 386 patients. There was no heterogeneity between the groups (I^2^=0), and a fixed effect model was used. The negative rate of tumor margin in the experimental group was higher than that in the control group, and the difference was statistically significant (OR=0.07, 95% CI=0.02-0.12, *P*=0.01) (Figure 7a).

##### 3.3.2.5 Hospitalization time

Five studies reported the stay time after surgery, so the final study included six studies and 365 patients. There was significant heterogeneity between the groups (I^2^=85%), so a random effects model was used. There was no significant difference in hospital stay between the two groups (MD=-2.12, 95% CI=-4.17- -0.08), *P*=0.04) (Figure 7b).

## 4. Discussion

Anatomical hepatectomy is one of the most ideal surgical methods for precise hepatectomy with the ability of accurately defining the range of liver sections. The main approach to mark the scope of resection in the anatomical right hemihepatectomy involves blocking the right branch of the portal vein and the right hepatic artery, marking the ischemic line on the liver surface, and cutting along the ischemic line 19-21. Then, the right branch of the puncture portal vein was injected with methylene blue, the right hemi liver was stained, and the resection margin was determined based on the staining results. However, in the actual process of hepatic parenchymal disconnection, it is difficult to distinguish the difference in color by utilizing the traditional approach described above, and also it is poor in liver cirrhosis or hepatic adhesion when the ischemic line is poor, because the boundary between the liver segments is not flat but rather an uneven and irregular plane 22, 23.

ICG is a near-infrared fluorescent dye that binds to plasma proteins and can be excited by external light with a wavelength of 750 to 810 nm, emitting near-infrared light at a wavelength of about 840 nm. It is received by PDE (photodynamic eye) and can be displayed in a developing device 24. In 2009, the ICG was first applied to HCC surgery 25-27, and then this technique was further applied to many other fields such as liver tumor detection, liver segmentation, biliary exploration and liver cell function assessment in transplanted liver transplantation. The corresponding hepatic segment (or hepatic lobe) portal vein branch (positive display method) or peripheral intravenous ICG (negative display method) can be used for three-dimensional visualization to gain better visualization of liver segment or hepatic lobe boundary. Intraoperative real-time guidance of accurate anatomical liver or lobe resection improved the accuracy of anatomical hepatectomy. In this study, we found that the liver function recovery faster in the guided group, which may be related to the absence of hepatic occlusion, which reduced the ischemia-reperfusion injury of the liver. Because of the precise navigation during surgery, the amount of bleeding is reduced, the guided group achieves zero blood transfusion, and reduced the risk of postoperative recurrence and metastasis due to blood transfusion.

In anatomical right hemihepatectomy, the application of FIGFI can not only display the boundaries between the right and left hemipatella on the surface of the liver, but also achieve a stronger contrast in the liver section to distinguish the right and left hemi livers. The liver parenchyma off-off plane can be corrected in real time. And its branches are naturally exposed and well preserved. The liver or lobe ICG fluorescence imaging methods mainly include positive display and reverse display. The positive display is suitable for the development of liver segments or sub-hepatic segments with less liver pedicles (1-2 branches). The fluorescence contrast is strong, but it is technically difficult and often requires puncture under ultrasound guidance 8, 9, 28. The anti-display method is applicable to the liver segments where portal vein branches are easily revealed, and is often developed for hepatic or hepatic segments with more liver pedicles (3 or more). In this study, the injection method was explored. The positive staining method requires the surgeon to have a higher puncture technique, which can be combined with the preoperative 3D imaging technique to determine the length of the right hepatic pedicle and the structure of the right branch of the portal vein to increase the success rate. The negative staining can reduce the time of liver rupture as it quickly stains, and is more convenient and easier to inject through the peripheral vein.

One of the patients originally intended to block the right portal vein and then developed the left hemi liver by negative visualization. Because the anatomical variation of the left and right branches of the portal vein was found to be difficult to completely separate during surgery, the author tried to let ICG enter the left hepatic blood through the left hepatic artery sinus. 1 ml (2.5 mg/ml) ICG was injected via the peripheral vein after ligation and blocking of the right hepatic artery and the main portal vein. ICG entered into the liver through the left hepatic artery 2 min later, and the left hepatic liver was successfully developed. Anatomical right hemihepatectomy was performed which is difficult to achieve via methylene blue staining. What we learned from this study is that right hemihepatectomy should be used to develop the left half liver anti-display method, because ICG fluorescence has a certain ability of liver tissue penetration, along the scope of the fluorescein resection, in theory, the remaining liver is slightly larger than the left liver. During the process, it is not easy to damage the trunk of the middle hepatic vein, and the hepatocytes and biliary tract that are fluorescently developed, avoid the influence of blood extravasation containing ICG on the determination of the fluorescence range.

Some studies have reported that the success rate of intraoperative anatomical liver resection fluorescence imaging reaches up to 95.8%^6^. In our previous operation, one patient was proposed to perform anti-display method during the operation. Due to the anatomical variation of the hepatic vessels, ligation of the right portal vein and the right hepatic artery after the ligation and separation of the portal vein, the right hemi liver was still visualized in the peripherally injected ICG. There are previous reports showing that staining failures occurred mostly in the anti-display method. When the liver segment had more supply of liver pedicles, it failed to block the entire hepatic pedicles, causing staining failure. The three-dimensional visualization technology can construct a three-dimensional model of the portal vein that is faithful to the patient's actual anatomy. For the anatomical abnormality of the portal vein in the right hemihepatectomy, it can be combined with three-dimensional visualization technology to achieve a 3-D display of the right hepatic portal vein, which helps the anatomical right hemi liver During the laparotomy, FIGFI cannot achieve continuous navigation. During the process of hepatic parenchyma off, it is necessary to close the shadowless lamp to detect the fluorescence to achieve real-time navigation. Therefore, the fusion of the operative field and fluorescence needs to be continuously converted, making the operation complicated and causing longer operation time. In addition, preoperative ICG injections may affect the hepatic cross-sectional fluorescence display when the tumor is closer to the incision margin. Preoperative removal of the preoperative liver 3-D reconstruction technique can avoid such occurrence. The combination of ICG fluorescence technology and preoperative three-dimensional reconstruction of the liver can be used to guide liver resection from three-dimensional morphological anatomy of liver tissue, which can further improve the accuracy of liver resection.

Some clinical centers have reported the use of ICG molecular fluorescence imaging technology for the diagnosis and treatment of liver tumors 3-6, 29. However, whether it can improve the surgical treatment of liver tumors is still lack of domestic and foreign high-quality evidence. This study also collected relevant literature, systematically evaluated the effectiveness and safety of ICG molecular fluorescence imaging technology in the accurate diagnosis and treatment of liver tumors, and provided reliable evidence-based medical evidence for its widespread clinical promotion.

But the study has the following limitations: (1) The number of included studies is small and the methodological quality is generally low. The main reason is that there is no RCT study of ICG molecular fluorescence imaging technology applied to liver tumors, only retrospective cohort study. (2) Most of the study samples are small, there are no detailed surgical resection records, and fewer cases of right hemihepatectomy. Meanwhile, there are few reports on long-term prognosis, which still needs to be compared in later studies. (3) Some outcome indicators have significant heterogeneity in the merger, and the subgroup analysis is less likely to be included due to the inclusion of the study, which may affect the credibility of the results. (4) Most of the literature is for Asian populations and may have an impact on the extrapolation of results.

In summary, in the treatment of HCC with right hemihepatectomy, intraoperative ICG three-dimensional visualization of the liver, FIGFI can be a long time, clearly shows the median right and left hepatic segmentation of the liver median fracture, real-time guidance of anatomical right hemihepatectomy treatment of HCC can help improve the accuracy of right hemihepatectomy for HCC. It has a profound impact in the era of precision liver surgery, because of the fast postoperative liver function recovery and the low recurrence rate.

## Figures and Tables

**Table 1:** Preoperative clinical features between ICG guided and traditional group

Characteristics		ICG Group	Traditional Group	*t*/χ^2^ value	*P* value
Gender	male	15	26	0.399	0.528
	female	3	3		
HBsAg	positive	12	21	0.175	0.675
	negative	6	8		
AFP (ng/ml)	≥400	7	15	0.735	0.391
	<400	11	14		
Age (years)		52.9±12.1	59.3±10.4	-1.866	0.071

**Table 2:** Preoperative liver function index between ICG guided and traditional group

Characteristics	ICG Group	Traditional Group	*t*/χ^2^ value	*P* value
ALT	34.9±18.4	42.2±26.8	-1.004	0.321
AST	34.4±17.3	42.4±15.1	-1.635	0.112
TB	14.8±3.9	15.9±6.5	-0.622	0.537
ALB	43.2±3.5	39.8±3.6	3.169	0.003
PLT	151.4±49.9	170.5±80.9	-0.896	0.375
PT	10.4±0.9	11.1±1.1	-2.042	0.047

**Table 3:** Intraoperative index between ICG guided and traditional group

Characteristics	ICG Group	Traditional Group	*t*/χ^2^ value	*P* value
Blood (ml)	261±144	438±384	-2.240	0.031
Blood transfusion (ml)	0	345±442	-4.201	<0.001
Tumor size (cm)	6.3±3.2	7.8±2.5	-1.784	0.081
Hepatic occlusion (min)	0	4.9±7.3	-2.835	0.007

**Table 4:** Postoperative index between ICG guided and traditional group

Characteristics	ICG Group	Traditional Group	t/χ2 value	P value
ALT	368.6±388.5	485.6±404.8	-0.978	0.333
AST	375.8±284.1	589.8±397.7	-2.147	0.037
TB	30.8±15.8	28.7±17.0	0.420	0.676
ALB	35.7±4.4	33.8±4.3	1.421	0.162
PT	14.0±1.4	13.9±2.1	0.221	0.826
Hospitalization day	11.1±7.8	13.4±7.1	-1.084	0.284
Cost	4.7±1.3	4.6±1.5	0.208	0.836

**Table 5:** Preoperative clinical features between negative and positive ICG guided group

Characteristics		negative staining	positive staining	*t*/χ^2^ value	*P* value
Gender	male	9	6	0.047	0.829
	female	2	1		
HBsAg	positive	9	3	2.922	0.087
	negative	2	4		
AFP (ng/ml)	≥400	4	3	0.0765	0.7831
	<400	7	4		
Age (years)		54.6±11.9	50.1±12.8	0.759	0.459

**Table 6:** Preoperative liver function index between negative and positive ICG guided group

Characteristics	negative staining	positive staining	*t* value	*P* value
ALT	40.3±20.3	27.0±12.3	1.516	0.149
AST	38.7±20.0	27.6±9.7	1.365	0.191
TB	15.7±3.3	13.5±4.7	1.142	0.270
ALB	43.6±3.5	42.5±3.5	0.695	0.497
PLT	142.2±46.8	166.0±54.9	-0.985	0.339
PT	10.6±1.1	10.2±0.7	0.660	0.519

**Table 7:** Intraoperative index between negative and positive ICG guided group

Characteristics	negative staining	positive staining	*t* value	*P* value
Blood (ml)	241±130	293±169	-0.736	0.473
Tumor size (cm)	6.5±3.3	6.1±3.1	0.273	0.788

**Table 8:** Postoperative index between negative and positive ICG guided group

Characteristics	negative staining	positive staining	*t* value	*P* value
ALT	418.8±482.3	289.7±167.5	0.676	0.509
AST	403.7±335.0	331.9±194.6	0.512	0.616
TB	27.7±12.1	35.6±20.5	-1.042	0.313
ALB	34.8±5.1	36.9±3.1	-0.961	0.351
PT	14.3±1.3	13.5±1.6	1.124	0.277
Hospitalization day	13.1±9.4	7.9±1.8	1.434	0.171
Cost (RMB)	4.9±1.4	4.3±1.0	1.003	0.331

**Table 9:** Basic characteristics of inclusion literature

Author	Time	Region	Case	Male	Lap Surgical	Mean size(cm)
Kaibori^11^	2011	Japan	52/50	76/26	0/0	5.3/5.0
Handgraaf^12^	2017	Netherlands	67/87	38/56	0/0	7.4/3.2
Nishino^6^	2017	Japan	23/29	19/17	0/0	-
Aoki^13^	2018	Japan	25/72	15/44	25/72	2.9/2.22
Chen^14^	2019	China	12/12	9/10	12/12	5.03/4.62
Zhou^15^	2019	China	21/21	15/15	21/21	3.1/3.2
Zhang^16^	2019	China	13/11	8/9	13/11	-
Yao^17^	2019	China	28/27	23/22	0/0	6/8
Xiao^18^	2019	China	67/46	35/22	67/46	2.44/2.8

## Abbreviations

HCC: Hepatocellular carcinoma
ICG: Indocyanine green
FIGFI: fusion ICG fluorescence imaging
ALT: Alanine aminotransferase
TB: Total albumin
PT: Prothrombin time
CT: Computed Tomography
RCT: Randomized controlled trial
OR: Ods ratio
MD: Mean difference
CI: Confidence interval
PDE: Photodynamic eye
